# Supplementary material for: Mapping the evolution of fertility support policies in China: A content and instrumental analysis
Source: PLoS One. 2025 Oct 9;20(10):e0332137. doi: 10.1371/journal.pone.0332137 (PMC12510515; doi:10.1371/journal.pone.0332137)
Supplement: S1 Appendix — (ZIP) [file pone.0332137.s001.zip › S1 Appendix. 226 original policy documents/187-国家发展改革委办公厅、民政部办公厅、国家卫生健康委办公厅关于编报积极应对人口老龄化工程和托育建设2021年中央预算内投资计划建议的通知(FBM-CLI-4-5016739).doc]

国家发展改革委办公厅、民政部办公厅、国家卫生健康委办公厅关于编报积极应对人口老龄化工程和托育建设2021年中央预算内投资计划建议的通知

**发布部门：** [国家发展和改革委员会(含原国家发展计划委员会、原国家计划委员会)](https://www.pkulaw.com/law/chl?Aggs.IssueDepartment=60301) [民政部](https://www.pkulaw.com/law/chl?Aggs.IssueDepartment=60208) [国家卫生健康委员会](https://www.pkulaw.com/law/chl?Aggs.IssueDepartment=60321) [机构沿革](javascript:void(0))

**发文字号：**发改办社会〔2021〕544号

**发布日期：**2021.07.06

**实施日期：**2021.07.06

**时效性：** [现行有效](https://www.pkulaw.com/law/chl?Aggs.TimelinessDic=01)

**效力级别：** [部门工作文件](https://www.pkulaw.com/law/chl?Aggs.EffectivenessDic=XE0303)

**法规类别：** [预算管理](https://www.pkulaw.com/law/chl?Aggs.Category=03126) [老少妇幼残保护](https://www.pkulaw.com/law/chl?Aggs.Category=01604)

国家发展改革委办公厅、民政部办公厅、国家卫生健康委办公厅关于编报积极应对人口老龄化工程和托育建设2021年中央预算内投资计划建议的通知
（发改办社会〔2021〕544号）

各省、自治区、直辖市及计划单列市、新疆生产建设兵团发展改革委、民政厅（局）、卫生健康委，黑龙江北大荒农垦集团有限公司：
　　为推进实施《“十四五”积极应对人口老龄化工程和托育建设实施方案》（发改社会〔2021〕895号，以下简称《实施方案》），2021年国家发展改革委安排中央预算内投资支持养老和托育服务体系设施建设。现将编报投资计划建议方案的有关要求通知如下：
　　**一、**总体要求
　　（一）计划建议方案必须符合《实施方案》及《积极应对人口老龄化工程和托育建设中央预算内投资专项管理办法》（发改社会规〔2021〕525号）相关要求，遴选前期工作成熟、具备开工条件的项目，确保投资计划下达后能尽快开工，不符合专项建设条件、前期工作不完备的项目一律不纳入安排范围。
　　（二）计划建议方案要按照《中央预算内投资计划编制管理暂行办法》《国家发展改革委关于加强政府投资项目储备编制三年滚动投资计划的通知》《国家发展改革委办公厅关于使用国家重大建设项目库加强项目储备编制三年滚动投资计划有关问题的通知》要求做好衔接，未纳入国家重大建设项目库的项目，不得列入本次建议方案。
　　（三）2021年投资要严格防范由此增加的地方政府债务风险。各地要统筹财力，避免重复安排，确保落实中央预算内投资项目的地方资金。对直接下达的项目，各地上报的投资计划建议方案文件中地方政府或地方行政主管部门的配套资金承诺函中需注明：“经认真审核，所报投资计划符合我省（区、市）财政承受能力和政府投资能力，不会造成地方政府隐性债务”。
　　（四）安排地方的中央预算内投资属于奖补性质，资金安排将与各地“一老一小”整体解决方案的编制和实施情况、培训疗养机构转型发展养老服务的进展和绩效挂钩。各地方对项目负有主体责任。要落实项目实施和监管责任，在中央预算内投资计划下达后，应明确每一个项目的项目（法人）单位及项目责任人、日常监管直接责任单位及监管责任人，及时开展调度。

　　**二、**工作安排
　　（一）请各地发展改革部门根据上述要求，会同民政、卫生健康部门按要求提出建议方案，上报国家发展改革委、民政部、国家卫生健康委。
　　（二）各地在编报建议方案过程中，地级行政区要同步编制“一老一小”整体解决方案，编制培训疗养机构转型发展养老服务实施方案，于2021年底前印发实施，由省级发展改革部门汇总后上报国家发展改革委备案。国家发展改革委将会同民政部、国家卫生健康委等部门，对“一老一小”整体解决方案实施情况适时开展评估。
　　（三）对于投资切块下达的项目，建议方案只需上报投资总需求等内容，不再单独上报单个具体项目情况及可研批复、项目真实性声明、配套资金承诺函等附件；对于投资直接下达的项目，请填写每个单体项目的建议方案，并提交项目可研批复、项目真实性声明、配套资金承诺函等单行材料附件。
　　（四）计划建议方案需使用重大建设项目库进行编报。各地要确保上报项目与通过重大建设项目库上报的三年滚动投资计划一致。拟申请补助的投资项目一定要选择“年度投资计划”并准确填写申请资金年份等内容。若未准确选择相关指标，将导致无法从库中遴选出相关项目。
　　（五）请各单位根据填报说明及时填报绩效表（每个省份填写一份，详见附件）。
　　（六）请各地抓紧开展工作，在2021年7月16日前，将年度申报项目通过国家重大建设项目库推送国家发展改革委，报送投资计划建议方案EXCEL电子版（见附件）和直接下达项目的单行材料；并同步报送2021年投资计划建议方案和所附单行材料等纸质正式文件。建议方案表必须从国家重大建设项目库的年度投资计划编报区选择“2021年中央预算内投资计划申报表”进行打印，不得更改，并将建议方案表EXCEL电子版以电子邮件形式分送国家发展改革委（社会发展司）、民政部（规划财务司）、国家卫生健康委（老龄健康司、人口监测与家庭发展司）。
　　联系方式：国家发展改革委社会发展司周建010-68501412，rkfzc_shs@ndrc.gov.cn，崔文通010-68502590；民政部规划财务司付晓媛010-58123312，mztj@mca.gov.cn；国家卫生健康委老龄健康司曹群010-62030676，caoqun@nhc.gov.cn，人口监测与家庭发展司李欣航010-62030920，rkjtsjtc@nhc.gov.cn。
　　附件：[1.积极应对人口老龄化工程和托育建设2021年中央预算内投资计划建议方案表](https://resources.pkulaw.cn/staticfiles/fagui/20210716/18/13/0/0fbb0efaf7e788f91c658772c4255f4f.pdf)
　　[2.积极应对人口老龄化工程和托育建设2021年中央预算内投资绩效目标表](https://resources.pkulaw.cn/staticfiles/fagui/20210716/18/13/0/bd804c354b7764c6af254d00a759200a.pdf)

国家发展改革委办公厅
　　民政部办公厅
　　国家卫生健康委办公厅
　　2021年7月6日

© 北大法宝：（[www.pkulaw.com](https://www.pkulaw.com/)) 专业提供法律信息、法学知识和法律软件领域各类解决方案。北大法宝为您提供丰富的参考资料，正式引用法规条文时请与标准文本核对。
欢迎查看所有[产品和服务](https://www.pkulaw.com/helps/2.html)。法宝快讯：[如何快速找到您需要的检索结果？](https://www.pkulaw.com/helps/70.html)[法宝](https://www.pkulaw.com/helps/69.html)V6有何新特色？

扫描二维码阅读原文

**原文链接：**<https://www.pkulaw.com/chl/96d2b189208da00dbdfb.html>
